# Long-term fertilization altered microbial community structure in an aeolian sandy soil in northeast China

**DOI:** 10.3389/fmicb.2022.979759

**Published:** 2022-09-07

**Authors:** Shiyu Zhang, Xue Li, Kun Chen, Junmei Shi, Yan Wang, Peiyu Luo, Jinfeng Yang, Yue Wang, Xiaori Han

**Affiliations:** ^1^College of Land and Environment, Shenyang Agricultural University, Shenyang, China; ^2^National Engineering Research Center for Efficient Utilization of Soil and Fertilizer Resources, Shenyang, China; ^3^Monitoring and Experimental Station of Corn Nutrition and Fertilization in Northeast Region, Ministry of Agriculture, Shenyang, China; ^4^Department of Foreign Languages, Shenyang Agricultural University, Shenyang, China

**Keywords:** bacterial community, fungal community, long-term fertilization, Illumina MiSeq sequencing, aeolian sandy soil

## Abstract

Soil microorganisms play crucial roles in nutrient cycling and determining soil quality and fertility; thus, they are important for agricultural production. However, the impacts of long-term fertilization on soil microbial community remain ambiguous due to inconsistent results from different studies. The objective of this study was to characterize changes in bacterial and fungal diversity and community structures after 12 years of different fertilization in aeolian sandy soil by analyzing 16S rRNA and ITS rRNA gene sequences and the soil properties to discover the driving factors. Eight different fertilizer treatments have been set up since 2009: no fertilizer (CK), chemical N fertilizer (N), chemical N and P fertilizer (NP), chemical N, P and K fertilizer (NPK), pig manure only (M), pig manure plus chemical N fertilizer (MN), pig manure plus chemical N and P fertilizer (MNP), pig manure plus chemical N, P, and K fertilizer (MNPK). The results indicated that the long-term application of chemical fertilizer reduced soil pH, whereas the addition of pig manure alleviated a decrease in soil pH value. Chemical fertilizer plus pig manure significantly improved soil available nutrients and soil organic carbon. Long-term MNPK fertilization resulted in changes in bacterial diversity due to effects on specific bacterial species; by contrast, all fertilization treatments resulted in changes in fungal diversity due to changes in soil properties. Principal component analysis indicated that fertilization had a significant effect on soil microbial community structure, and the effect of chemical fertilizer combined with pig manure was greater than that of chemical fertilizer alone. Soil available phosphorus, total phosphorus, and pH were the most important factors that influenced bacterial taxa, whereas soil pH, total phosphorus, organic carbon, ammonium nitrogen and nitrate nitrogen were the most important factors influencing fungal taxa after 12 years of fertilization in aeolian sandy soil.

## Introduction

Soil microorganisms are the crucial drivers of soil organic matter formation and decomposition (Zhang et al., 2012), and play critical roles in maintaining soil health, productivity and sustainability (Chaparro et al., 2012; Su et al., 2015), as well as in regulating crop production or disease suppression (Van Der Heijden et al., 2008; Berg, 2009). Even though microorganisms account for only a small part of soil quality, they play an essential role in the cycling of soil carbon (C), nitrogen (N), phosphorus (P), and sulfur (S), and are important components in maintaining soil quality and fertility (Wei et al., 2019; Zhang et al., 2019). Changes in soil microbial community also affect the productivity and function of agroecosystems. Due to the importance of microorganisms in agroecosystems, researchers have paid increasing attention to the study of soil microorganisms in recent years. Soil microbial communities are affected by human activities such as fertilization and tillage (Ding et al., 2017). Previous studies have shown that soil microorganisms could be influenced by many factors, such as soil type (Nicolitch et al., 2016), tillage system (Hartmann et al., 2015; Wang et al., 2020), fertilizer regime (Wang et al., 2021), sampling period (Wang et al., 2018), and crop species (Li et al., 2021).

Fertilization is an important agricultural practice to improve soil fertility and increase crop yield in agroecosystems (Francioli et al., 2016; Hu et al., 2017). Fertilization can indirectly impact soil microorganisms by changing soil properties or directly by input of nutrients (Pan et al., 2020; Yan et al., 2021). A previous study indicated that long-term application of chemical fertilizer resulted in a significant decline in soil bacterial diversity due to a decrease in soil pH value, while the addition of manure effectively alleviated this decline (Sun et al., 2015). However, some studies yielded contradictory results. For instance, some studies revealed that application of organic fertilizer could increase soil microbial diversity compared with application of chemical fertilizer, whereas others indicated that application of organic fertilizer could reduce microbial diversity (Hu et al., 2018; Xu et al., 2020; Yang et al., 2020). Soil pH is generally considered to be the main factor affecting soil bacterial diversity, while fungal diversity was generally less affected by soil pH (Rousk et al., 2010). Zhou et al. (2016) indicated that long-term chemical N and P fertilization reduced the soil fungal diversity and altered the fungal community distribution in a black soil. Sun et al. (2016) revealed that the application of organic matter significantly reduced the relative abundance of potentially pathogenic fungi and changed the soil fungal community structure. The responses of soil microbial community to fertilization have been reported widely (Hu et al., 2017; Yu et al., 2019; Mei et al., 2021). Understanding the changes in soil microbial communities under different fertilization regimes in different soils is needed for determining the optimal fertilization methods to improve soil conditions and crop productivity.

Aeolian sandy soils have poor soil structure, low organic matter and nutrient content, water leakage and poor fertilizer retention ability. Fertilization is an effective method for improving aeolian sandy soils. Although the effects of long-term inorganic fertilizer application combined with organic fertilizer on soil microbial communities have been investigated frequently, few studies have been reported on aeolian sandy soils, and the potential links between soil properties and microbial communities remain unclear in aeolian sandy soils in northeast China. Therefore, this study aimed to investigate the changes in soil microbial diversity and community structure in an aeolian sandy soil due to differential fertilization for 12 years and to determine the relationship between soil microbial diversity and community structure and soil properties.

High-throughput sequencing was used to analyze soil microbial diversity and community structure in the aeolian sandy soil after 12-year fertilization. Our objectives were: (i) to evaluate the effects of various fertilization regimes on soil microbial diversity and community structure in the aeolian sandy soil, and (ii) to discover the most important factors affecting soil microbial communities. We hypothesized that the combined application of chemical fertilizer and pig manure would be more favorable than chemical fertilizer or pig manure alone in improving soil nutrient status and enhancing soil microbial diversity, and 12-year fertilization would change soil microbial community structure by altering soil properties.

## Materials and methods

### Site description and experimental design

The long-term fertilization experiment was established in 2009 at the Soil and Fertilizer Test Base of the National Peanut Industry Technology System in Shenyang Agricultural University, Liaoning Province, northeastern China (40°48′N, 123°33′E). This region has a temperate sub-humid continental climate with annual precipitation 574∼684 mm. The frost-free period is 164 days, and the average annual temperature is 7.0∼8.1°C. The field experiment was a continuous cropping system of peanut. The tested soil is aeolian sandy soil, which is classified as Entisols according to the USDA Soil Taxonomy classification systems.

Eight treatments were arranged in a randomized block design in triplicate under the peanut continuous cropping system. Each plot was 2 × 1 m in size and separated by 0.2 m wide and 2 m deep cement walls. The treatments included: (1) control treatment (CK); (2) chemical N fertilizer (N); (3) chemical N and P fertilizer (NP); (4) chemical N, P, and K fertilizer (NPK); (5) pig manure only (M); (6) pig manure plus chemical N fertilizer (MN); (7) pig manure plus chemical N and P fertilizer (MNP); (8) pig manure plus chemical N, P, and K fertilizer (MNPK). Urea, calcium superphosphate and potassium sulfate were applied as chemical fertilizers to supply N, P, and K, respectively. Fertilizer application rates of the eight treatments are shown in Supplementary Table 1. The average nutrient contents in pig manure were as follows: 7.2 g⋅kg^–1^ N; 8.7 g⋅kg^–1^ P_2_O_5_; 10.0 g⋅kg^–1^ K_2_O; and 83.5 g⋅kg^–1^ organic C. The field was plowed (20 cm deep) before planting. The whole process (including hoeing, ridging, fertilizing, sowing, weeding, and harvesting) was done manually.

Peanuts (*Arachis hypogaea* L.) were sown on May 12 and harvested on September 20, 2020. The width of the ridge was 60 cm; two rows of peanuts were planted in each ridge and the distance between two rows was 30 cm. Two seeds were sown in each hole, and the distance between two holes was 14 cm. The planting density of peanut was 30,000 plants ha^–1^.

### Sample collection and soil property measurements

Rhizosphere soil samples were collected during the peanut harvest in 2020. Eight peanut plants were randomly selected in each plot, and the whole plants with their roots were dug out with a spade (approximately 20 cm depth). The roots were shaken gently to remove the loosely adhering soil and carefully brushed to collect the remaining rhizosphere soil. The visible plant roots and residues were removed from fresh rhizosphere soil samples, sieve (<2 mm) and divided into three subsamples. One subsample was air-dried to determine soil properties; the second subsample was used for determining soil ammonium nitrogen (NH_4_^+^-N) and soil nitrate nitrogen (NO_3_^–^-N), and the third subsample was stored at –80°C for deoxyribonucleic acid (DNA) extraction.

Soil pH was measured using a pH meter (Mettler-Toledo 320) with a 1:2.5 ratio of soil to water. Total nitrogen (TN) and soil organic carbon (SOC) were determined by an Element Auto-Analyzer (Vario MAX CN; Elementar, Hanau, Germany). Soil ammonium nitrogen (NH_4_^+^-N) and nitrate nitrogen (NO_3_^–^-N) were extracted with 1 M KCl and measured by an autoanalyzer (AutoAnalyzer3, SEAL Analytical, Germany). Available phosphorus (AP) and available potassium (AK) was extracted using 0.5 M NaHCO_3_ solution and 1 M NH_4_OAc, respectively, and determined by molybdenum-blue colorimetry and flame photometry (M410, Sherwood Scientific Ltd., United Kingdom), respectively (Bao, 2000). Soil total phosphorus (TP) and total potassium (TK) was determined according to Lu (1999).

### Soil deoxyribonucleic acid extraction, polymerase chain reaction amplification, and Illumina sequencing

Soil DNA was extracted using a Powersoil^®^ DNA isolation kit (Mo Bio Laboratories, Inc., Carlsbad, CA, United States) according to the manufacturer’s instructions. The concentration and purity of extracted DNA were assessed by a NanoDrop ND-1000 (NanoDrop Technologies Inc., Wilmington, DE, United States).

The hypervariable region V3-V4 of the bacterial 16S ribosomal RNA (rRNA) gene was amplified with the primer pair 338F (5′-ACTCCTACGGGAGGCAGCAG-3′) and 806R (5′-GGACTACHVGGGTWTCTAAT-3′) (Xu et al., 2016). The 20 μL polymerase chain reaction (PCR) reaction mixture contained 4 μL of 5 × FastPfu Buffer, 2 μL of dNTPs (2.5 mM), 0.2 μL of bovine serum albumin (BSA), 0.8 μL of forward and reverse primers (5 μM), 0.4 μL of FastPfu DNA polymerase, 10 ng of template DNA, and was adjusted with ddH_2_O to 20 μL. The fungal ITS rRNA genes were amplified with the primer pair ITS1F (5′-CTTGGTCATTTAGAGGAAGTAA-3′) and ITS2R (5′-GCTGCGTTCTTCATCGATGC-3′) (Adams et al., 2013). The 20 μL PCR reaction mixture contained 2 μL of 10 × Buffer, 2 μL of dNTPs (2.5 mM), 0.2 μL of BSA, 0.8 μL of forward and reverse primers (5 μM), 0.2 μL of rTaq DNA Polymerase, 10 ng of template DNA, and was adjusted with ddH_2_O to 20 μL. The PCR thermal cycle conditions were: an initial denaturation step at 95°C for 3 min, followed by 27 cycles (for bacteria) or 35 cycles (for fungi) of denaturing at 95°C for 30 s, annealing at 55°C for 30 s, extension at 72°C for 45 s, and a final extension at 72°C for 10 min. The purified amplicons were sequenced on an Illumina MiSeq PE300 platform (Illumina, San Diego, CA, United States) by Majorbio Bio-Pharm Technology Co., Ltd. (Shanghai, China). The raw sequence data were deposited in the NCBI Sequence Read Archive database with the SRA accession numbers of PRJNA838988 and PRJNA839049 for bacteria and fungi, respectively.

### Statistical analyses

Statistical analyses were performed using SPSS 19.0 (SPSS Inc., Chicago, IL, United States). The differences between treatments were analyzed by one-way analysis of variance (ANOVA), followed by Least Significant Difference (LSD) test. Spearman’s correlation analysis was used to evaluate the relationship between soil microbial diversity and soil properties. The online Circos software was used to perform the Circos graphs for bacterial and fungal community compositions.^1^ Principal coordinates analysis (PCoA) was performed to analyze beta-diversity in different treatments based on Bray-Curtis distance. Permutational multivariate analysis of variance (PERMAVONA) was calculated using the R package “vegan” to determine the significant differences in beta-diversity among the treatments. The linear discriminant analysis effect size (LEfSe) method was used to estimate potential microbial markers under different treatments. Redundancy analysis (RDA) with Monte Carlo permutation tests (*n* = 999) and Mantel test were performed using R to analyze the relationship between soil microbial communities and soil properties.

## Results

### Soil properties

The changes in soil properties under various treatments after 12 years of fertilization are shown in Table 1. Overall, fertilization increased the contents of SOC, TN, TP, NH_4_^+^-N, NO_3_^–^-N, AP, and AK (*P* < 0.05), except the N treatment. The contents of SOC, TN, TP, NO_3_^–^-N, AP, and AK were significantly higher in manure treatments (M, MN, MNP, and MNPK) than in chemical fertilizer treatments (N, NP, and NPK) (*P* < 0.05). The soil pH varied from 5.34 to 6.18. Long-term chemical fertilizer application significantly decreased the soil pH (*P* < 0.05), whereas the application of pig manure effectively alleviated soil acidification (*P* < 0.05).

**TABLE 1:** Soil properties for different fertilizer treatments.

Treatment	SOC	TN	TP	TK	NH_4_^+^-N	NO_3_^–^-N	AP	AK	pH
								
	(g⋅kg^–1^)	(g⋅kg^–1^)	(g⋅kg^–1^)	(g⋅kg^–1^)	(mg⋅kg^–1^)	(mg⋅kg^–1^)	(mg⋅kg^–1^)	(mg⋅kg^–1^)	
CK	5.66 ± 0.03e	0.46 ± 0.01f	0.21 ± 0.01e	24.58 ± 0.13bc	4.39 ± 0.34b	3.69 ± 0.10e	9.32 ± 0.34d	66.82 ± 2.93e	5.87 ± 0.03c
N	5.99 ± 0.10e	0.52 ± 0.01ef	0.20 ± 0.01e	24.62 ± 0.51bc	5.56 ± 0.11a	4.71 ± 0.21de	9.81 ± 0.34d	66.93 ± 1.99e	5.34 ± 0.02f
NP	6.78 ± 0.19d	0.62 ± 0.01d	0.29 ± 0.01d	24.31 ± 0.51c	5.71 ± 0.24a	5.55 ± 0.41d	22.73 ± 0.87c	72.72 ± 2.61d	5.53 ± 0.02e
NPK	6.20 ± 0.03de	0.55 ± 0.01de	0.29 ± 0.01d	27.09 ± 0.67a	3.78 ± 0.28c	4.51 ± 0.41de	25.30 ± 1.81c	155.85 ± 0.69c	5.73 ± 0.04d
M	10.83 ± 0.01b	1.05 ± 0.02b	0.41 ± 0.01c	26.69 ± 0.12a	3.93 ± 0.23c	14.86 ± 0.98a	52.94 ± 1.46a	163.31 ± 2.32b	6.18 ± 0.02a
MN	10.93 ± 1.12b	1.08 ± 0.12ab	0.45 ± 0.01c	25.03 ± 0.55bc	4.41 ± 0.19b	10.61 ± 0.78b	46.37 ± 1.73b	160.02 ± 0.66bc	6.07 ± 0.03b
MNP	11.87 ± 0.34a	1.14 ± 0.03a	0.61 ± 0.03a	25.38 ± 0.91b	3.59 ± 0.04c	9.40 ± 0.32c	56.77 ± 2.36a	160.15 ± 4.36bc	6.12 ± 0.03b
MNPK	9.76 ± 0.24c	0.95 ± 0.04c	0.56 ± 0.05b	27.48 ± 0.52a	3.84 ± 0.31c	9.82 ± 0.77bc	56.11 ± 5.47a	205.25 ± 1.69a	6.07 ± 0.03b

Values are means (n = 3) ± SD (standard deviation). Values within the same column followed by different lowercase letters indicate significant differences at P < 0.05. SOC, soil organic carbon; TN, total nitrogen; TP, total phosphorus; TK, total potassium; NH_4_^+^-N, ammonium nitrogen; NO_3_^–^-N, nitrate nitrogen; AP, available phosphorus; AK, available potassium. CK, no fertilizer; N, chemical N fertilizer; NP, chemical N and P fertilizer; NPK, chemical N, P, and K fertilizer; M, pig manure only; MN, pig manure plus chemical N fertilizer; MNP, pig manure plus chemical N and P fertilizer; MNPK, pig manure plus chemical N, P, and K fertilizer.

### Soil bacterial and fungal alpha-diversity

A total of 745,702 bacterial and 1,313,236 fungal sequences were detected in the 24 soil samples (eight treatments in biological triplicates) and were clustered into 5804 and 1541 operational taxonomic units (OTUs) based on 97% sequence similarity. The OTUs were assigned to 31 phyla and 849 genera for bacteria, and 17 phyla and 443 genera for fungi. The rarefaction curves approached saturation with the increasing number of sequences (Supplementary Figure 1), indicating that the depth of sequencing was sufficient to characterize the soil microbial community in this study.

Long-term fertilization resulted in variations in alpha-diversity of rhizosphere soil microbial community, including species richness [ACE (Abundance-based Coverage Estimator) indices] and diversity (Shannon indices) (Table 2). Soil bacterial ACE index was highest in the MN treatment and lowest in the NPK treatment. The ACE index was higher in the manure treatments (M, MN, MNP, and MNPK) than the chemical fertilizer treatments (N, NP, and NPK). Correlation analysis showed that bacterial ACE index was significantly positively correlated with SOC and TN (*P* < 0.01), and was positively correlated with TP, AP, and pH (*P* < 0.05, Table 3). Soil bacterial Shannon index was higher in the CK, M, and MN compared with the other treatments, and was lowest in the MNPK treatment.

**TABLE 2:** Soil microbial alpha-diversity indices for different treatments.

Treatment	Bacteria		Fungi	
		
	ACE	Shannon	ACE	Shannon
CK	3467.57 ± 115.95ab	6.57 ± 0.08a	585.42 ± 55.15bc	3.25 ± 0.18c
N	3358.03 ± 78.86b	6.40 ± 0.07bc	538.31 ± 38.21c	3.19 ± 0.10c
NP	3502.98 ± 194.8ab	6.50 ± 0.11ab	620.39 ± 22.42ab	3.21 ± 0.11c
NPK	3333.66 ± 118.57b	6.42 ± 0.09bc	637.62 ± 24.06ab	3.66 ± 0.12ab
M	3524.10 ± 114.92ab	6.57 ± 0.09a	671.34 ± 19.29a	3.48 ± 0.32bc
MN	3682.70 ± 19.62a	6.57 ± 0.02a	615.21 ± 55.14ab	3.44 ± 0.18bc
MNP	3579.42 ± 84.67ab	6.47 ± 0.04abc	678.45 ± 40.19a	3.89 ± 0.12a
MNPK	3573.72 ± 305.81ab	6.36 ± 0.05c	665.86 ± 6.18a	3.96 ± 0.13a

Values are means (n = 3) ± SD (standard deviation). Values within the same column followed by different lowercase letters indicate significant differences at P < 0.05.

**TABLE 3:** Spearman correlation coefficient between soil properties and soil microbial alpha-diversity.

		SOC	TN	TP	TK	NH_4_^+^-N	NO_3_^–^-N	AP	AK	pH
Bacteria	ACE index	0.551**	0.569**	0.445*	–0.087	–0.083	0.394	0.411*	0.330	0.408*
	Shannon index	0.170	0.188	–0.051	–0.290	0.171	0.196	–0.107	–0.030	0.289
Fungi	ACE index	0.578**	0.571**	0.663**	0.439*	-0.594**	0.534**	0.735**	0.691**	0.733**
	Shannon index	0.510*	0.505*	0.730**	0.516**	-0.693**	0.366	0.735**	0.656**	0.503*

Significance are demonstrated as: P < 0.05 (*) (two tailed), P < 0.01 (**) (two tailed). SOC, soil organic carbon; TN, total nitrogen; TP, total phosphorus; TK, total potassium; NH_4_^+^-N, ammonium nitrogen; NO_3_^–^-N, nitrate nitrogen; AP, available phosphorus; AK, available potassium. Values in bold indicate significant correlations.

Regarding soil fungal community diversity, the ACE index was lowest in the N treatment and highest in the M treatment. Compared with the CK treatment, ACE indices increased in the other fertilization treatments, except the N treatment. Correlation analysis showed that soil fungal ACE index was negatively correlated with NH_4_^+^-N (*P* < 0.01), and was positively correlated with other soil properties (Table 3). The Shannon index was highest in the MNPK treatment and lowest in the N treatment. The Shannon indices were higher in the manure treatments (M, MN, MNP, and MNPK) than the other treatments, except the NPK treatment. The soil fungal Shannon index was negatively correlated with NH_4_^+^-N (*P* < 0.01), and was positively correlated with the other soil properties, except NO_3_^–^-N (Table 3).

### Soil bacterial and fungal community composition and structure

**FIGURE 1:**
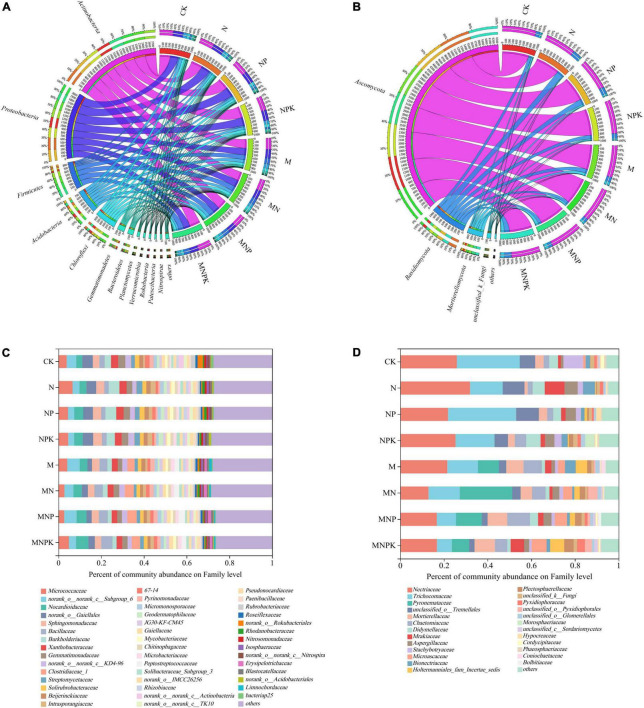
Relative abundances of the rhizosphere microbial community composition on phylum and family levels under different treatments. **(A)** Bacterial community on phylum level. **(B)** Fungal community on phylum level. **(C)** Bacterial community on family level. **(D)** Fungal community on family level. CK, no fertilizer; N, chemical N fertilizer; NP, chemical N and P fertilizer; NPK, chemical N, P, and K fertilizer; M, pig manure only; MN, pig manure plus chemical N fertilizer; MNP, pig manure plus chemical N and P fertilizer; MNPK, pig manure plus chemical N, P, and K fertilizer.

Twelve bacterial phyla with the relative abundance >1% were detected in all soil samples, including *Actinobacteria, Proteobacteria, Firmicutes, Acidobacteria, Chloroflexi, Gemmatimonadetes, Bacteroidetes, Rokubacteria, Planctomycetes, Verrucomicrobiota, Nitrospirae*, and *Patescibacteria* (Figure 1A). The most dominant phylum was *Actinobacteria* (29.98–38.40%), followed by *Proteobacteria* (22.40–30.85%) and *Firmicutes* (4.50–17.84%). The relative abundances of *Actinobacteria* and *Proteobacteria* in the no-manure treatments (CK, N, NP, NPK) were higher than those in the manure treatments (M, MN, MNP, and MNPK), whereas the relative abundance of *Firmicutes* in the no-manure treatments was significantly lower than that in the manure treatments (*P* < 0.05; Supplementary Figure 2A). The relative abundances of *Gemmatimonadetes, Rokubacteria*, and *Nitrospirae* decreased with fertilization. No statistically significant differences among the treatments were detected in the relative abundances of *Actinobacteria, Chloroflexi, Planctomycetes*, and *Verrucomicrobiota.* For fungal communities, three phyla and one unidentified phylum with the relative abundance > 1% were detected, including *Ascomycota* (71.43–84.13%), *Basidiomycota* (8.95–20.41%) and *Mortierellomycota* (3.71–9.79%) (Figure 1B). The relative abundance of *Ascomycota* was lowest in the MNPK treatments. The relative abundance of *Basidiomycota* was higher in the all fertilization treatments (except MN) compared with the CK treatment. The relative abundance of *Mortierellomycota* was lowest in the N treatment, and was significantly higher in the treatments with than without pig manure (Supplementary Figure 2B).

The soil bacterial and fungal compositions at the family level are detailed in Figure 1. For bacteria, the relative abundances of *norank_o__Gaiellales, Streptomycetaceae* (belonging to *Actinobacteria*), *Sphingomonadaceae*, and *Burkholderiaceae* (belonging to *Proteobacteria*) in the chemical fertilizer treatments were significantly higher than those in the manure treatments. The relative abundances of *Bacillaceae* and *Clostridiaceae_1* (belonging to *Firmicutes*) increased significantly in the manure treatments (Figure 1C). Regarding fungal families, the relative abundances of *Nectriaceae, Trichocomaceae, Didymellaceae, Stachybotryaceae* (belonging to *Ascomycota*) and *unclassified_o__Tremellales* (belonging to *Basidiomycota*) were higher in the chemical fertilizer treatments compared with those that received pig manure. The relative abundances of *Pyronemataceae, Chaetomiaceae, Microascaceae* (belonging to *Ascomycota*) and *Mortierellaceae* (*Mortierellomycota*) increased significantly in the manure treatments (Figure 1D).

**FIGURE 2:**
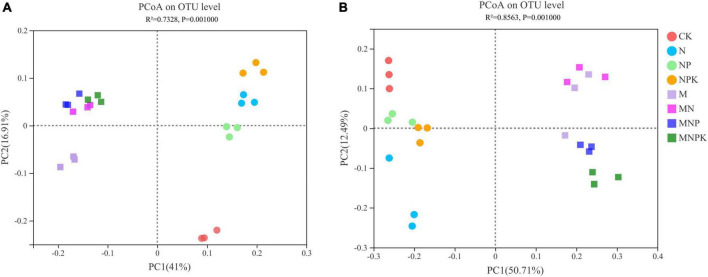
Principal coordinate analysis (PCoA) of **(A)** bacterial community and **(B)** fungal community.

The variations in soil bacterial and fungal communities under different treatments were assessed by principal coordinate analysis (PCoA) based on Bray-Curtis distance measure (Figure 2). The first two principal components (PC1 and PC2) together explained 57.91% of variation in bacterial community and 63.20% of variation in fungal community. Soil bacterial and fungal communities were divided into two groups, whereby manure treatments (M, MN, MNP, and MNPK) represented one group, and no manure treatments (CK, N, NP, and NPK) were clustered into the other group. The PERMANOVA analysis also demonstrated significant differences among the treatments regarding soil bacterial and fungal community compositions (*P* < 0.01; Supplementary Table 2).

### Taxonomic biomarkers of soil bacterial and fungal communities

**FIGURE 3:**
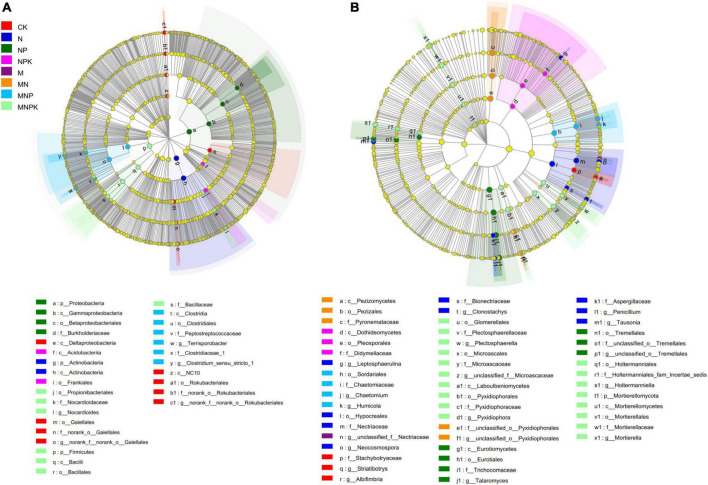
The linear discriminant analysis effect size (LEfSe) showed the significantly different taxa of bacterial communities **(A)** and fungal communities **(B)** under different treatments. The threshold of LDA score was 4.0.

The LEfSe was performed to identify high-dimensional biomarker taxa of soil bacterial and fungal communities with significantly different abundances among different treatments (Figure 3). In total, 29 bacterial and 50 fungal differentially abundant taxa were identified with an LDA threshold of 4.0. For the bacterial communities, *Gaiellales* (from class to its genus *norank_f__norank_o__Gaiellales*), *Deltaproteobacteria* class, and *Rokubacteria* (from phylum to its genus *norank_f__norank_o__Rokubacteriales*) were significantly higher in the CK treatment compared with all other treatments. *Actinobacteria* and *Proteobacteria* were the most enriched bacterial phyla in the N and NP treatments, respectively. The *Acidobacteriia* class within *Acidobacteria* and the *Frankiales* order within *Actinobacteria* were significantly enriched in the NPK treatment. The *Clostridia* class (from class to its genera *Clostridium_sensu_stricto_1* and *Terrisporobacter*) within *Firmicutes* was significantly enriched in the MNP treatment. The *Firmicutes* (from phylum to its family *Bacillaceae*) and *Propionibacteriales* (from order to its genus *Nocardioides*) within *Actinobacteria* were significantly enriched in the MNPK treatment (Figure 3A).

Regarding the fungal communities, genera *Albifimbria* and *Striatibotrys* within *Ascomycota* were significantly enriched in the CK treatment. *Hypocreales* (from order to its genera *Clonostachys* and *Neocosmospora*), *Aspergillaceae* (from family to its genus *Penicillium*), *Leptosphaerulina* within *Ascomycota*, and *Tausonia* within *Basidiomycota* were significantly enriched in the N treatment. *Eurotiomycetes* (from class to its genus *Talaromyces*) within *Ascomycota* and *Tremellales* within *Basidiomycota* were significantly enriched in the NP treatment. *Dothideomycetes* and *Pezizomycetes* (both *Ascomycota*) were enriched in the NPK and MN treatments, respectively. *Sordariales* (from order to its genera *Chaetomium* and *Humicola*) were significantly higher in the MNP treatment. Only the MNPK treatment had a phylum-level marker, namely *Mortierellomycota*. Some taxa within *Ascomycota* and *Basidiomycota* were significantly enriched in the MNPK treatment (Figure 3B).

### Relationships between soil microbial communities and soil properties

**FIGURE 4:**
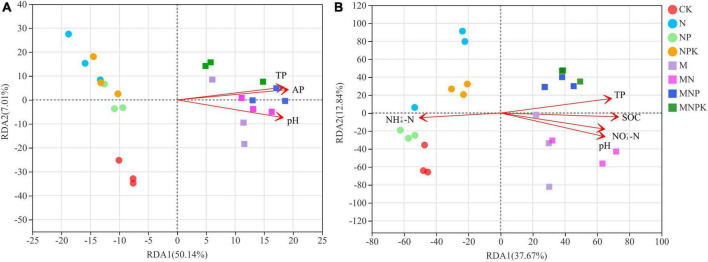
Redundancy analysis (RDA) of the relationship between microbial communities and the soil properties. **(A)** Bacterial community, **(B)** fungal community. SOC, soil organic carbon; TP, total phosphorus; NH_4_^+^-N, ammonium nitrogen; NO_3_^–^-N, nitrate nitrogen; AP, available phosphorus.

To assess how soil properties impacted soil bacterial and fungal community structure in different treatments, Spearman’s correlations using the Mantel test (Table 4) between soil microbial communities and soil properties, and RDA (Figure 4) were performed. The Mantel test results showed soil bacterial community was significantly correlated with SOC, TN, TP, AP, and pH, and soil fungal community was significantly correlated with SOC, TN, TP, NH_4_^+^-N, NO_3_^–^-N, AP, AK, and pH. The soil properties included in RDA were selected on the basis of their collinearity and strength of their correlations with the soil microbial community structure by VIF < 20 (Lin et al., 2018) and significance (≤0.05) in Monte Carlo permutation test. The first two axes of the RDA explained 57.15 and 50.51% of the total variance in the bacterial and fungal communities, respectively. For the bacterial communities, the most important influencing factors were TP, AP, and pH (Figure 4A). For the fungal communities, the most important influencing factors were pH, SOC, NH_4_^+^-N, NO_3_^–^-N, and TP (Figure 4B).

**TABLE 4:** Mantel analysis of the relationships between the soil microbial community structure and soil properties.

	Bacteria		Fungi	
		
	*r*	*P*	*r*	*P*
SOC	0.220	**0.008**	0.191	**0.015**
TN	0.248	**0.004**	0.209	**0.011**
TP	0.264	**0.002**	0.323	**0.001**
TK	–0.025	0.617	0.034	0.241
NH_4_^+^-N	0.080	0.113	0.207	**0.007**
NO_3_^–^-N	0.113	0.113	0.248	**0.003**
AP	0.268	**0.004**	0.251	**0.006**
AK	0.008	0.058	0.165	**0.020**
pH	0.248	**0.004**	0.435	**0.000**

Boldface numbers indicate that p-values were significant at the 0.05 level. SOC, soil organic carbon; TN, total nitrogen; TP, total phosphorus; TK, total potassium; NH_4_^+^-N, ammonium nitrogen; NO_3_^–^-N, nitrate nitrogen; AP, available phosphorus; AK, available potassium.

**FIGURE 5:**
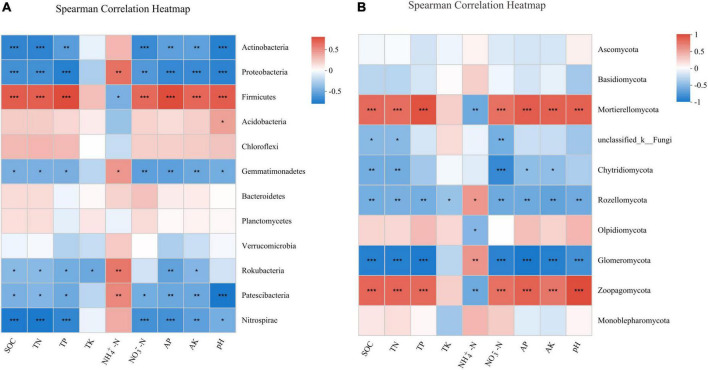
Spearman’s correlation analysis between soil properties and the relative abundance of bacterial **(A)** and fungal **(B)** phylum under different treatments. *P < 0.05; **P < 0.01; ***P < 0.001.

The Spearman’s correlation analysis between the soil bacterial community and soil properties showed that the relative abundances of *Actinobacteria* and *Proteobacteria* were significantly negatively correlated with SOC (*P* < 0.001), TN (*P* < 0.001), TP (*P* < 0.01), NO_3_^–^-N (*P* < 0.01), AP (*P* < 0.01), AK (*P* < 0.01), and pH (*P* < 0.001). By contrast, the relative abundance of *Firmicutes* was significantly positively correlated with SOC (*P* < 0.001), TN (*P* < 0.001), TP (*P* < 0.001), NO_3_^–^-N (*P* < 0.001), AP (*P* < 0.001), AK (*P* < 0.001), and pH (*P* < 0.001, Figure 5A). For fungal community, the Spearman’s correlation analysis indicated that the relative abundance of the two most dominant phyla *Ascomycota* and *Basidiomycota* did not correlate with soil properties. Nevertheless, the relative abundance of *Mortierellomycota* had significant positive correlations with SOC (*P* < 0.001), TN (*P* < 0.001), TP (*P* < 0.001), NO_3_^–^-N (*P* < 0.001), AK (*P* < 0.001), AP (*P* < 0.001), and pH (*P* < 0.001), and a significant negative correlation with NH_4_^+^-N (*P* < 0.01, Figure 5B).

## Discussion

### Long-term fertilization improved soil fertility

As showed in Table 1, the contents of the major soil nutrients (N, P and K) and soil organic carbon were increased after the 12-year application of fertilizers. The contents of SOC, TN, TP, NO_3_^–^-N, AP, and AK were significantly higher in the manure treatments than in the chemical fertilizer treatments (*P* < 0.05), indicating that the effects of pig manure alone or combined with chemical fertilizer on improving soil fertility and accelerating soil nutrient turnover were more significant than those of chemical fertilizer alone, which was in line with previous studies (Wei et al., 2017; Pan et al., 2020). The 12-year chemical fertilization significantly decreased soil pH (*P* < 0.05), and the soil pH value was lowest in the N treatment. These findings were unsurprising because the ammonium-based N fertilizers (such as urea) are known to acidify soils with poor pH buffering capacity (typical for sandy soils). However, pig manure could effectively alleviate soil acidification (Bei et al., 2018); in the present study, the M treatment had the highest pH value. Pig manure is enriched with organic matter and nutrients including nitrogen, phosphorus, and potassium (Ding et al., 2017; Li et al., 2022); thus our results indicated general improvements in soil fertility after 12 years of combined application of pig manure and chemical fertilizers on aeolian sandy soil.

### Effects of fertilization on soil bacterial and fungal diversity

Many previous studies showed that the application of manure alone or combined with chemical fertilizer could enhance soil bacterial diversity (Shen et al., 2015; Chen et al., 2016). In the present study, the ACE indices of bacterial abundance were increased in the manure treatments, which was in line with previous study (Chaudhry et al., 2012). This might be due to the fact that manure application enhanced the availability of carbon substrates in soil and promoted root exudation (Bittman et al., 2005). The bacterial ACE indices were positively correlated with SOC and TN, which also confirmed the above explanation. However, soil bacterial Shannon index was lowest in the MNPK treatment in the present study, followed by the N treatment (Table 2). It might have been due to the disappearance of many bacterial taxa with the application of manure, resulting in a decrease in diversity. The 12-year application of chemical nitrogen fertilizer significantly decreased bacterial diversity in the rhizosphere soil, which might be related to the effects of soil acidification caused by soil N enrichment on microorganisms (Geisseler and Scow, 2014). In addition, nitrogen levels could affect some physiological characteristics of plants, thereby altering root exudates or signals and thus altering diversity of rhizosphere microorganisms (Basal and Szabó, 2018). Contrary to predictions, although fertilization changed soil bacterial diversity, there was no significant correlation between bacterial diversity and soil properties.

A previous study found that long-term organic fertilizers, especially manure, could decrease soil fungal diversity (He et al., 2008). However, Ding et al. (2017) found that inorganic fertilizer combined with manure could weakly improve soil fungal diversity. In our study, the fungal ACE indices were increased in all fertilization treatments except N treatment. The Shannon index was increased in all fertilization treatments except N and NP treatments. Manure application led to higher fungal diversity than chemical fertilization (Table 2). On the one hand, differential results in different studies are likely due to diverse soil types. On the other hand, it might be the application of manure stimulated the growth of fungi. The application of manure provides organic substances, and fungi, as indispensable decomposers, could digest complex organic compounds into bioavailable forms (Xiang et al., 2020). The soil fungal Shannon index was lowest in the N treatment in our study, which was in line with the conclusion that chemical nitrogen fertilizer reduced fungal biodiversity (Zhou et al., 2016). A significant decreasing trend of soil fungal diversity with increasing NH_4_^+^-N (*R*^2^ = –0.693, *P* < 0.01) and decreasing pH (*R*^2^ = 0.503, *P* < 0.05) was revealed by Spearman correlation analysis (Table 3). The 12-year application of nitrogen fertilizer significantly reduced soil pH value and increased soil inorganic nitrogen (Table 1). The decrease of soil pH might have been associated with a reduction or disappearance of some fungal taxa due to diminishment of the original habitat suitable for the growth of fungi (Zhou et al., 2016), while the enrichment of nitrogen affected the adaptability of fungi, especially arbuscular mycorrhizal fungi, to the soil environment (Zhang et al., 2021). In contrast to no correlation between the bacterial Shannon index and soil properties, the fungal Shannon index was positively correlated with a range of soil properties (i.e., SOC, TN, TP, TK, AP, AK), indicating that long-term fertilization had a greater impact on rhizosphere soil fungal diversity than bacterial diversity in aeolian sandy soil.

### Alterations in soil microbial community composition and structure

Soil microorganisms play an important role in soil nutrient turnover, which in turn affects soil microbial community. In this study, 12-year fertilization significantly affected soil microbial community composition (Figure 1). Twelve bacterial phyla were detected in soil samples, of which two phyla (*Actinobacteria* and *Proteobacteria*) dominated in abundance in all treatments, indicating that these bacterial phyla have strong adaptability to the environment. The relative abundance of *Actinobacteria* and *Proteobacteria* decreased with the application of manure, whereas that of *Firmicutes* increased in the manure treatments. The relative abundances of *Actinobacteria* and *Proteobacteria* were significantly negatively correlated with SOC, TN, TP, NO_3_^–^-N, AP, AK, and pH. By contrast, the relative abundance of *Firmicutes* was significantly positively correlated with these soil properties (Figure 5). *Actinobacteria* have high tolerance to environmental stress and can survive in low-pH soil environments (Dai et al., 2018). Therefore, the relative abundance of *Actinobacteria* was highest in the N treatment, and *Actinobacteria* was the indicator bacterial taxon for the N treatment (Figure 3A), which was consistent with other studies of *Actinobacteria* in the rhizosphere soil (e.g., Dai et al., 2018). Regarding different survival strategies in bacterial communities, *Proteobacteria* and *Firmicutes* are copiotrophic bacteria (Li et al., 2019). *Proteobacteria* are closely related to the C and N cycles, and *Firmicutes* express glycosylated enzymes involved in hydrolysis of cellulose and chitin (Wegner and Liesack, 2016). However, the relative abundance of *Proteobacteria* was negatively correlated with soil organic carbon content in our study (Figure 5), which might have been caused by the oligotrophic characteristics of its dominant family *Burkholderiaceae* (Sun et al., 2015; Purkamo et al., 2016), in the present study, the relative abundance of *Burkholderiaceae* decreased with the application of manure (Figure 1C). Phylogenetic network analysis showed that *Firmicutes* were negatively correlated with *Actinobacteria* and *Proteobacteria* at the family level (Supplementary Figure 3). With the application of manure, *Firmicutes* proliferated, which might have been another reason for the decrease of the relative abundance of *Actinobacteria* and *Proteobacteria* in the manure treatments (Mei et al., 2021). The members of *Firmicutes* were indicator bacteria of the MNP and MNPK treatments. *Bacillaceae* were the most abundant family of *Firmicutes* in the MNPK treatment, and they are well known to contribute to plant growth and health in numerous ways (Tu et al., 2018; Lee et al., 2021), indicating that long-term application of manure enhanced the microbial community of aeolian sandy soil.

For fungal communities, we detected three predominant phyla in soil samples, i.e., *Ascomycota, Basidiomycota*, and *Mortierellomycota.* In this study, the relative abundance of *Ascomycota* was highest in all treatments, and its members were the indicator fungi in each treatment (Figure 3B), which was consistent with Yan et al. (2021). *Ascomycota* are considered organic decomposers that generally increase in abundance with organic fertilizers application (Ye et al., 2020). However, in the present study, *Ascomycota* and *Basidiomycota* were found to have no correlation with soil properties. The relative abundance of *Mortierellomycota* was increased in the manure treatments and was significantly positively correlated with SOC, TN, TP, NO_3_^–^-N, AK, AP, and pH, indicating long-term application of pig manure could increase soil nutrient contents and provide a more suitable habitat for *Mortierellomycota*. Therefore, as saprophytic fungi, *Mortierellomycota* benefitted from the application of manure (Cong et al., 2020). Hence, *Mortierellomycota* (from phylum to genera) were indicator fungal taxa of the MNPK treatment. Narisawa et al. (1998) found that *Mortierella* were involved in plant pathogen inhibition, alleviating the incidence and severity of Chinese cabbage bulb disease and Fusarium wilt and root rot in banana. The present study proved again that long-term application of chemical fertilizer combined with manure was beneficial to the microbial community of aeolian sandy soil.

The PCoA revealed that fertilization significantly changed soil microbial community structure (Figure 2). Soil bacterial and fungal community compositions were different in the manure treatments and non-manure treatments, which was in agreement with Pan et al. (2020). The RDA results showed that soil properties had a large impact on the changes in soil bacterial and fungal community composition (Figure 4), indicating that these soil properties played a decisive role in the soil microbial community structure. Soil bacteria community structure was significantly influenced by soil properties, such as AP, TP, and pH (Figure 4A). Spearman’s correlation analysis demonstrated that most bacteria were associated with these soil properties (Figure 5A). Similarly, a range of soil properties (i.e., pH, TP, SOC, NH_4_^+^-N, and NO_3_^–^-N) contributed to shaping the fungal community structure (Figure 4B). In this study, soil TP and pH were the main driving factors influencing both bacterial and fungal communities. Phosphorus is an important nutrient for the growth of bacteria and fungi and can directly influence the composition and functioning of soil microbial community (Richardson et al., 2011; Liao et al., 2018). Many studies have shown that fungi generally grow optimally over a wide pH range and are therefore less affected by soil pH than bacteria (Rousk et al., 2010; Zhalnina et al., 2015). Contrary to these claims, the soil pH was the main environmental factor driving fungal community changes in our study. This might have been due to a significant positive correlation between the major fungal phylum *Mortierellomycota* and soil pH. Bradley et al. (2006) found N fertilization caused significant changes in the composition of soil fungal communities. Zhang et al. (2021) showed that an appropriate amount of N could improve the efficiency of mycorrhizal symbiosis. In our study, soil fungal community was affected by soil carbon and nitrogen, which is consistent with the saprophytic status of most fungi (Liu et al., 2015; Ding et al., 2017). To sum up, different fertilization methods altered soil microbial community composition by varying soil properties.

## Conclusion

The contents of soil available nutrients and soil organic carbon were higher in the manure treatments compared with those that received chemical fertilizer only. Chemical fertilizer combined with manure increased the diversity of fungi. Long-term fertilization changed soil microbial community structure by altering soil properties. Soil TP, AP, and pH were the most important factors that influenced bacterial taxa, whereas soil pH, SOC, NH_4_^+^-N, NO_3_^–^-N, and TP were the most important factors influencing fungal taxa after 12-year fertilization in an aeolian sandy soil.

## Data availability statement

The datasets presented in this study can be found in online repositories. The names of the repository/repositories and accession number(s) can be found in the article/Supplementary material.

## Author contributions

SZ: conceptualization, methodology, investigation, data curation, writing – original draft and review and editing. XL, KC, and JS: investigation and data curation. YW: linguistic modification. PL and JY: visualization and funding acquisition. YuW: investigation, data curation, and supervision. XH: conceptualization, writing – review and editing, supervision, project administration, funding acquisition, and resources. All authors reviewed the manuscript.

## Footnotes

1
http://circos.ca/
