# Supplementary material for: Long-term fertilization altered microbial community structure in an aeolian sandy soil in northeast China
Source: Front Microbiol. 2022 Sep 7;13:979759. doi: 10.3389/fmicb.2022.979759 (PMC9490088; doi:10.3389/fmicb.2022.979759)
Supplement: Supplementary file 1 [file Data_Sheet_1.PDF]

Supplementary Material

Long-term fertilization altered microbial community structure in an aeolian sandy soil in northeast China

Shiyu Zhang[1,2,3], Xue Li[1,2,3], Kun Chen[1,2,3], Junmei Shi[1,2,3], Yan Wang[4], Peiyu Luo[1,2,3], Jinfeng Yang[1,2,3], Yue Wang[1,2,3]* and Xiaori Han[1,2,3]*

[1] College of Land and Environment, Shenyang Agricultural University, Shenyang, China

[2] National Engineering Research Center for Efficient Utilization of Soil and Fertilizer Resources, Shenyang, China

[3] Monitoring & Experimental Station of Corn Nutrition and Fertilization in Northeast Region, Ministry of Agriculture, Shenyang, China

[4] Department of Foreign Language, Shenyang Agricultural University, Shenyang, China

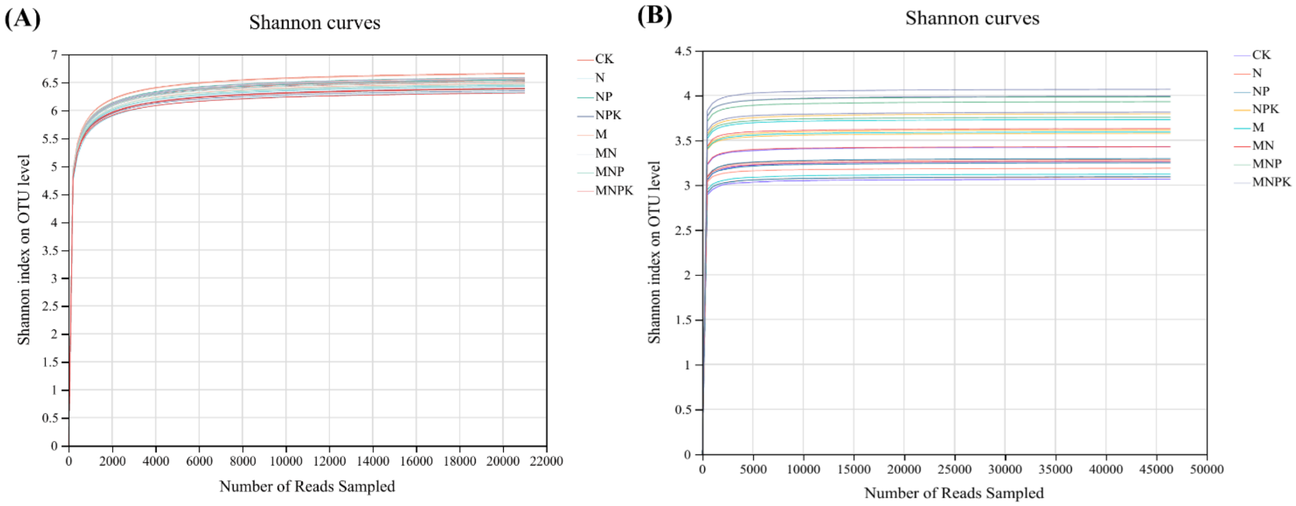

Supplementary Figure 1. Rarefaction Curves of soil bacterial (**A**) and fungal community (**B**). Abbreviations: CK, no fertilizer; N, chemical N fertilizer; NP, chemical N and P fertilizer; NPK, chemical N, P and K fertilizer; M, pig manure only; MN, pig manure plus chemical N fertilizer; MNP, pig manure plus chemical N and P fertilizer; MNPK, pig manure plus chemical N, P and K fertilizer.

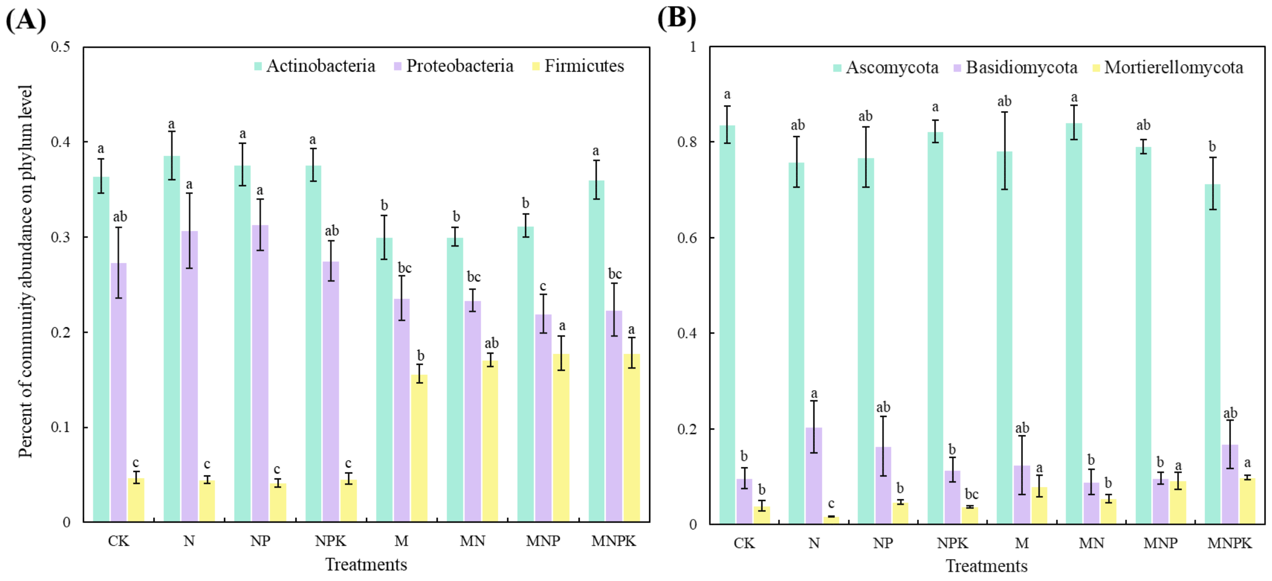

Supplementary Figure 2. The relative abundances of predominant soil bacterial (**A**) and fungal (**B**) communities at the phylum level.

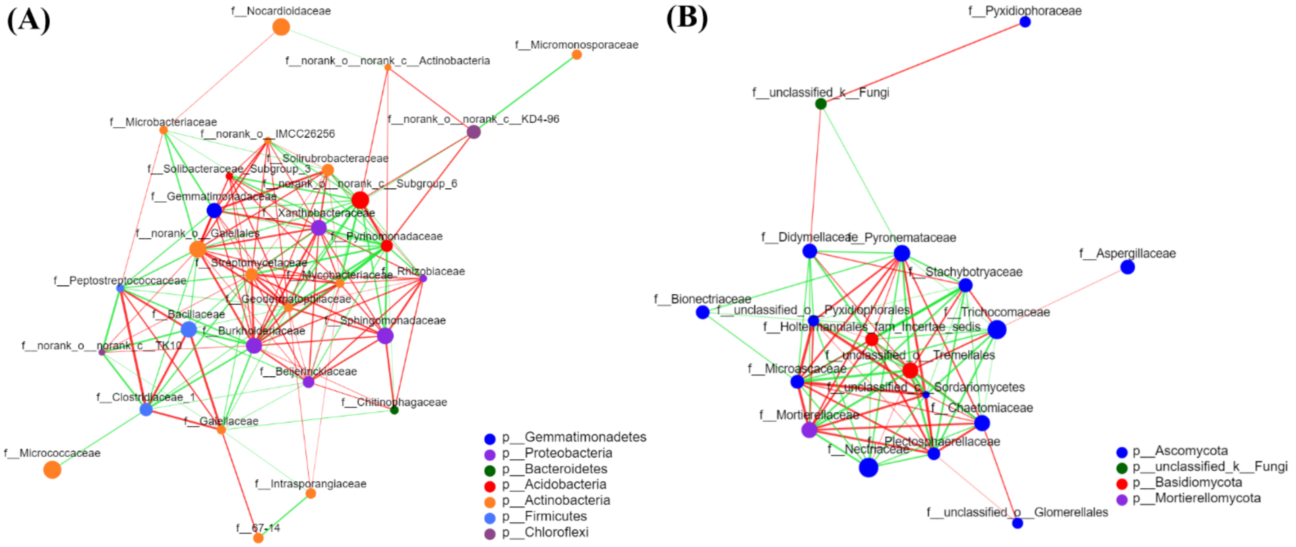

Supplementary Figure 3. Molecular ecological networks of bacterial (**A**) and fungal (**B**) communities based on correlation analysis. A green edge indicates a negative interaction between two individual nodes, while a red edge indicates a positive interaction.
