# Supplementary material for: Long-term fertilization altered microbial community structure in an aeolian sandy soil in northeast China
Source: Front Microbiol. 2022 Sep 7;13:979759. doi: 10.3389/fmicb.2022.979759 (PMC9490088; doi:10.3389/fmicb.2022.979759)
Supplement: Supplementary file 2 [file Table_1.DOCX]

Supplementary Material

**Long-term fertilization altered microbial community structure in an aeolian sandy soil in northeast China**

**Shiyu Zhang^1,2,3^, Xue Li^1,2,3^, Kun Chen^1,2,3^, Junmei Shi^1,2,3^, Yan Wang^4^, Peiyu Luo^1,2,3^, Jinfeng Yang^1,2,3^, Yue Wang^1,2,3*^ and Xiaori Han^1,2,3*^**

^1^ College of Land and Environment, Shenyang Agricultural University, Shenyang, China

^2^ National Engineering Research Center for Efficient Utilization of Soil and Fertilizer Resources, Shenyang, China

^3^ Monitoring & Experimental Station of Corn Nutrition and Fertilization in Northeast Region, Ministry of Agriculture, Shenyang, China

^4^ Department of Foreign Language, Shenyang Agricultural University, Shenyang, China

**Table S1 Application rates of fertilizer in different treatments.**

| Treatment | Chemical fertilization rates (kg/hm^2^) | | | Pig manure(t/hm^2^) |
| --- | --- | --- | --- | --- |
|  | N | P_2_O_5_ | K_2_O |  |
| CK | 0 | 0 | 0 | 0 |
| N | 60 | 0 | 0 | 0 |
| NP | 60 | 75 | 0 | 0 |
| NPK | 60 | 75 | 105 | 0 |
| M | 0 | 0 | 0 | 13.5 |
| MN | 60 | 0 | 0 | 13.5 |
| MNP | 60 | 75 | 0 | 13.5 |
| MNPK | 60 | 75 | 105 | 13.5 |

Note: The long-term fertilization started since 2009, the cropping system was peanut continuous cropping. Abbreviations: CK, no fertilizer; N, chemical N fertilizer; NP, chemical N and P fertilizer; NPK, chemical N, P and K fertilizer; M, pig manure only; MN, pig manure plus chemical N fertilizer; MNP, pig manure plus chemical N and P fertilizer; MNPK, pig manure plus chemical N, P and K fertilizer.

**Table S2. The results of PERMANOVA analysis on microbial community structure at OTU level under grouping characteristics.**

| **Characteristics** | **Sums of Sqs** | **Mean Sqs** | **F_Model** | **R^2^** | ***P*_value** |
| --- | --- | --- | --- | --- | --- |
| **Bacteria** |  |  |  |  |  |
| Treatments | 1.087 | 0.155 | 6.270 | 0.733 | 0.001 |
| **Fungi** |  |  |  |  |  |
| Treatments | 2.083 | 0.298 | 13.625 | 0.856 | 0.001 |

Note: The PERMANOVA analysis were conducted based on Bray-Curtis; Treatments: CK, N, NP, NPK, M, MN, MNP, MNPK.
